# Study of the Photocatalytic Degradation of Highly Abundant Pesticides in Agricultural Soils

**DOI:** 10.3390/molecules27030634

**Published:** 2022-01-19

**Authors:** Mohamed H. EL-Saeid, Amal BaQais, Mashael Alshabanat

**Affiliations:** 1 Chromatographic Analysis Unit, Soil Science Department, College of Food & Agricultural Sciences, King Saud University, P.O. Box 2460, Riyadh 11451, Saudi Arabia; elsaeidm@ksu.edu.sa; 2 Department of Chemistry, College of Science, Princess Nourah Bint Abdulrahman University, Riyadh 11564, Saudi Arabia; mnalshbanat@pnu.edu.sa

**Keywords:** pesticides, residue, soil, photocatalytic degradation

## Abstract

Organic pesticides are major sources of soil pollution in agricultural lands. Most of these pesticides are persistent and tend to bio accumulate in humans upon consumption of contaminated plants. In this study, we investigate different natural soil samples that were collected from agricultural lands. The samples revealed the presence of 18 pesticides that belong to four different groups including organochlorines (OCP), organophosphorus (OPP), carbamates (Carb), and pyrethroids (Pyrth). The photocatalytic degradation of the five most abundant pesticides was studied in the presence and absence of 1% TiO_2_ or ZnO photocatalysts under UV irradiation at a wavelength of 306 nm. The five abundant pesticides were Atrazine (OCP), Chlorpyrifos methyl (OPP), Dimethoate (OPP), Heptachlor (OCP), and Methomyl (Carb). The results showed that photolysis of all pesticides was complete under UV radiation for irradiation times between 64–100 h. However, both photocatalysts enhanced photocatalytic degradation of the pesticides in comparison with photolysis. The pesticides were photocatalytically degraded completely within 20–24 h of irradiation. The TiO_2_ photocatalyst showed higher activity compared to ZnO. The organochlorine heptachlor, which is very toxic and persistent, was completely degraded within 30 h using TiO_2_ photocatalyst for the first time in soil. The mechanism of photocatalytic degradation of the pesticides was explained and the effects of different factors on the degradation process in the soil were discussed.

## 1. Introduction

Plants are indispensable sources of food for all living things. They are the sole producers in the food chains that are consumed by various consumers such as herbivorous animals or directly by humans. Therefore, maintaining uncontaminated plants is essential for the health of not only humans, but also for the entire ecosystem. Pesticides are widely used as an effective method to increase crops and protect them from “pests”.

The term pesticide means “pest-killer”. It is a substance or mixture of substances used for controlling, preventing, destroying or reducing the number of pests [1]. Pests are one of major problems that agriculture faces. They are defined as being any type of organism that is harmful, destructive, or annoying to humans and plants. They can destroy crops and cause diseases to humans, animals, or plants [2]. The most common pests are insects, unwanted plants, microorganisms such as fungi or bacteria, and rodents. Currently, pesticides are classified based on three criteria: the pest organism they kill, the route of entry, and the active ingredient of their chemical structure [3]. Based on the pest they control, pesticides are classified into four main groups: insecticides, fungicides, herbicides, and rodenticides. Based on the mode of entry, pesticides are mainly classified into systemic and non-systemic pesticides. Plants or animals absorb systemic pesticides [4].

Insecticides are classified based on the chemical composition of the active ingredients. Insecticides are classified into organochlorines pesticides (OCP), organophosphates pesticides (OPP), carbamates (CARB), and pyrethroids (PYTH).

Organochlorines (OCP), as their name suggests, contain chlorine atoms in addition to carbon and hydrogen. They are non-selective and they kill a wide range of beneficial and non-beneficial insects [4]. The most common OCPs include dichlorodiphenyl trichoroethane (DDT) and its derivatives (DDE and DDD) in addition to benzene hexachloride (BHC) [5,6,7], dicofol [8], and heptachlor [9], and endosulfan [10]. The major hazard of this class is manifested by its persistence and long-term residual effects in the environment. It is non-biodegradable, may last in the soil, plants, and animal tissues, and bioaccumulate in humans [4,5].

The main advantage of organophosphates (OPP) over OCPs is being biodegradable and consequently causing minimal environmental pollution [11]. However, they possess higher toxicity [12]. The most common examples include chlorpyrifos, chlorpyrifos methyl [13], dimethoate [14], methidathion [15], parathion, malathion, diaznon and glyphosate [16].

Carbamates are structurally similar to organophosphates but they originate from carbamic acid. They work in a similar manner to organophosphates, causing nerve poisoning which leads to paralysis and death [3]. They are biodegradable and are easy to degrade using natural environmental conditions. The most-often used carbamates include carbaryl, methomyl, carbofuran, propoxur, and aminocarb [16].

Finally, pyrethins and pyrethoids are interesting classes of organic pesticides characterized by their low persistence in soil [17]. They are biodegradable with limited half-lives. Synthetic pyrethoids are also commonly used pesticides. Their structure offers them enhanced stability and therefore more persistent residues than the natural pyrethrins. They are considered among the safest pesticides to be used in agriculture and farming [4].

Despite the importance of pesticides in saving crops, they can cause soil, water, and air pollution. The severity of harmful effects on human health depends on the route of exposure, the dose, and the length of exposure [18]. Consequently, there are two types of toxicity, acute and chronic. Acute toxicity results from single exposure to high dose of the pesticide and chronic toxicity results from long-term exposure to small, repeated doses of the pesticide for years or even decades [4,18,19,20]. Consuming contaminated food leads to bioaccumulation of the non-biodegradable pesticides in the human body. This causes chronic illness in humans and indirect effects such as birth defects, genetic modification, nervous disorders, cancers, and reproduction diseases [21]. Pesticides lead to fatalities of 5000–20,000 people and the poisoning of 500,000 to 1 million people every year [22]. Half of the intoxicated are farmers and agricultural workers while the other half are poisoned through food. Therefore, degrading pesticide residues is highly crucial. Several approaches have been reported for pesticide degradation in water such as oxidation, adsorption [23], biological treatment [24], photocatalysis [25], and coagulation [26]. Photocatalysis remains the most advanced and suitable technique for pesticide treatment due to its simplicity and sustainability [27]. Since the first report by Honda and Fujishima [28], several metal oxide semiconductors have been reported to act as heterogeneous photocatalysts for removing organic contaminants such as oxides of (Cu, Mn, Co, Cr, V, Ti, Bi, and Zn) [29]. Zinc oxide (ZnO) and titanium oxides (TiO_2_) are characterized by several interesting physical properties such as high refractive index, ultraviolet (UV) absorption, and dielectric constants. They are photoactive with excellent incident photoelectric conversion efficiency, are chemically stable, have high photo stability, and they possess a long-term corrosion resistance [30]. In addition, they are non-toxic, green, and cheap [31]. These properties render them as suitable candidates for various applications, especially degradation of chemicals in the water and in the air.

In our work, we are concerned with photodegradation of the five pesticides summarized in Table 1, particularly organochlorines due to their persistence in the soil and due to their nature of being systemic insecticides. This work investigates the presence of pesticide residues in soil samples of dates and vegetables in natural soils. Treatment of the five most abundant pesticides with highest concentrations was studied. Moreover, the photolysis of the pesticides was performed in absence of catalyst and photocatalytic degradation using 1% TiO_2_ and 1% ZnO as photocatalysts for pesticide degradation under UV radiation at wavelength of 306 nm.

The degradation of pesticides in aqueous solutions is widely studied [25], however there are limited studies on the degradation in soil. Our work presents an important and vital study for the photodegradation of dangerous pesticides in the soil. To the best of our knowledge, this is the first Saudi Arabian report that investigates treating soil samples using photocatalysis under UV radiation using photocatalysts such as TiO_2_ and ZnO.

## 2. Materials and Methods

### 2.1. Chemicals

TiO_2_ (Sigma-Aldrich Chemie GmbH, Germany (molecular weight: 79.87, CAS Number: 1317-80-2, 637,262 nanopowder, <100 nm particle size, 99.5% trace metals basis ZnO (Sigma-Aldrich Chemie GmbH, Germany (molecular weight: 81.39, CAS Number: 1314-13-2, 544,906 nanopowder, <100 nm particle size.

Residue-analysis grade solvents were used for the extraction and analysis. They include methanol, dichloromethane, hexane, acetone, and acetonitrile. They were purchased from Fisher Scientific (Fair Lawn, NJ, USA) with purity about 99.9%.

### 2.2. Soil Samples

Ten different samples of dates and vegetables were collected from different date palm and vegetable farms located in the Al-Kharj governorate, situated southeast of Riyadh in Saudi Arabia. Al-Kharj is a coastal area characterized by arid weather with an annual rainfall of 132 mm. Samples were collected from a soil depth up to 30 cm. The types of soil in this area are aridisol, entisol, saline, and calcareous.

### 2.3. Pesticide Standards

Twenty-one pesticide standards were investigated and utilized. They were provided by AccuStandard, 153 Inc., New Haven, CT, USA, with purity of 98–99.8%, either as individual (50 mg) or as combination values at a concentration of 100 μg·mL^−1^. The pesticides that were investigated are listed as active ingredient (group abbreviation). They include chlorpyrifos methyl (OPPs), dimethoate (OPPs), atrazine (HERB), and methomyl (CARB).

### 2.4. Soil Samples Collection and Preparation

The survey procedure of pesticide residues collection was accomplished in ten selected sites in the Al-Kharj region. The collected samples were subjected to remediation using UV/TiO_2_ and ZnO in soil. Five soil samples were collected from different locations in separate sections. For each site, three different areas were carefully screened to collect the soil samples (1 kg for each replicate) from the outside layer (0–30 cm depth) and subsurface layer (30–60 cm depth). The collected soil samples were carefully placed on a clean plastic sheet and and judiciously handled on the place using a small spade.. Then, the collected samples were air-dried and sieved through a 2 mm sieve to remove any impurities. The spiked soil samples were prepared by the addition of the suitable amounts of the spiking solution (5 mg/L^−1^ of pesticides in acetone) to a 10 g soil sample. Before analysis, the spiked soil samples were allowed to settle and waited about 30 min until the solvent has completely evaporated. Finally, the soil samples were extracted by means of QuEChERS and then analyzed by GC-MS/MSTQD.

### 2.5. Extraction and Cleanup of Multi-Pesticide Residue by QuEChERS

To extract the target pesticides, a 10 g soil sample was introduced into a 50 mL centrifuge tube and mixed with 8 mL deionized water. The obtained mixture was vortexed and allowed to hydrate for 25–30 min, and then 12 mL of acetonitrile was added to respective samples. The samples were continuously shaken for 5 min to extract pesticide residues. The contents of QuEChERS original extraction solution (2 g MgSO_4_ and 1 g of NaCl) was added to a portion of soil sample in the centrifuge tube. Samples were immediately vortexed for at least 1 min and then centrifuged (≥3500 rcf) for 5 min. Then, an aliquot sample of supernatant (1.8 mL) was transferred to a 2 mL QuEChERS C-18 SPE tube. Samples were vortexed again for 2 min and centrifuged for another 2 min at high rcf (e.g., ≥5000). Finally, pesticides present in the extracted soil samples were analyzed by gas chromatography–mass spectrometry (GC–MS-TIC) [32]. QuEChERS kits and SPE tubes were purchased from Phenomenex, Madrid Avenue, Torrance, CA, USA.

### 2.6. Pesticide Analysis by Gas Chromatography–Mass Spectrometry (GC–MS-TIC)

A gas chromatography–mass spectrometry (GC–MS) was utilized for steps of analyte separation, detection, and identification. The measurement was performed on an Agilent (Palo Alto, CA, USA) 6890 N gas chromatograph equipped with an Agilent DB-5MS column (30 m × 0.25 mm × 0.25 µm film thickness) and 5973 N mass selective detector (Table 2) according to EL-Saeid et al. 2021 [32].

### 2.7. Remediation by Photolysis Degradation Experiment

For the pesticide remediation, the photolysis of the five pesticide residues was investigated, including chlorpyrifos methyl, dimethoate, atrazine, heptachlor, and methomyl. The photolysis was performed for different durations under UV irradiation at 306 nm using Boekel UV Crosslinker (BUV) model: 234100-2: 230 VAC, 175 W, 0.8 A, (Boekel Scientific, 855 Pennsylvania Blvd. Feasterville, PA, USA). The distance between UV lamps and soil samples was kept constant at 15 cm, and the UV irradiation intensity was 1071 µWcm^−2^.

### 2.8. Soil Sample Treatments

The treated samples were chosen to be the highly contaminated, i.e., those with highest concentration of the five pesticide residues described above. A total of 10 g of each soil sample was incubated in petri dishes (9 cm diameter) for 32 h under UV light/306 nm. Three petri dishes were removed every two hours and the remaining quantity of each tested pesticide was measured. A similar procedure was applied in the presence of photo catalysts (1% of TiO_2_ and 1% of ZnO) under UV remediation for 2 to 32 h.

### 2.9. QAQC Strategies

Quality control samples were prepared and analyzed in duplicate samples; blank and spiked, and/or certified reference material (CRM) purchased for this purpose and processed with each batch (5–10 samples) of sample. For each compound in the group of pesticides, the QuEChERS and GC-MS or GC-MSMS/TSQ 8000 method limit of detection (LOD), limit of quantification (LQD), repeatability, reproducibility, accuracy, and precession were determined.

## 3. Results and Discussion

The investigation of the pesticide residues in the soils of palms and vegetables revealed the presence of 18 pesticide residues. These pesticides are summarized and categorized based on their chemical structures in Supplementary Materials in Table S1.

Pesticide residues were detected belonging to the four classes described earlier. Seven types of organophosphates were found: chlorpyrifos methyl, dimethoate, primiphos-methyl, chlorpyrifos, methidathion, ethion and diazinon. Four types of organochlorines were detected: dicofol, heptachlor, endosulfan, and the herbicide atrazine. It is important to mention that the banned p,p-DDT organochlorine insecticide and its derivatives p,p-DDE, p,p-DDD were not detected in the soil samples. Two pesticides, which are carbamates, were found, i.e., methomyl and carbaryl, in addition to the pyrethoids deltamethrin, cypermethrin, permethrin, and β- cyfluthrin. The fungicide carbendazim was also detected.

**Figure 1:**
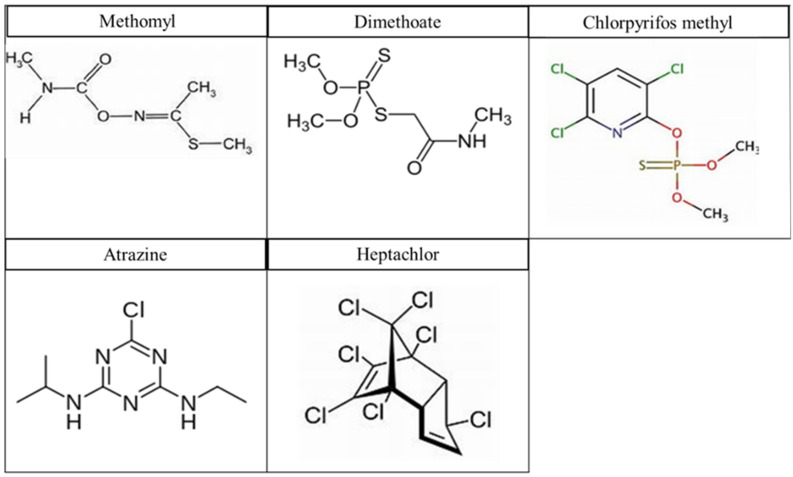
The chemical structures of the five pesticides.

The pesticides with highest concentrations in the soil were atrazine (Herb), chlorpyrifos methyl (OPP), dimethoate (OPP), heptachlor (OCP), and methomyl (Carb). The chemical structures of the pesticides are shown in Figure 1. The photoremediation of these five pesticide residues in soil were studied in absence (photolysis) and presence of two photocatalysts zinc oxide (ZnO) and titanium oxide (TiO_2_) under UV radiation at wavelength λ = 306 nm.

**Figure 2:**
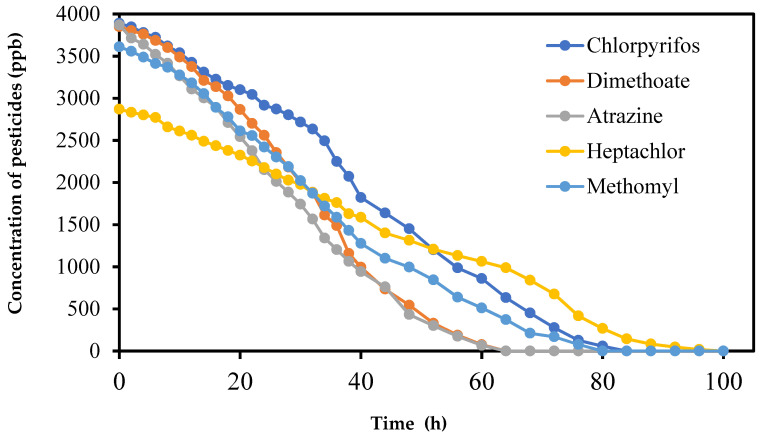
Variation of the concentration of the pesticides as a function of time under UV irradiation in absence of photocatalysts.

Figure 2 shows the photodegradation results of the five pesticides in absence of photocatalyst at λ = 306 nm. The largest decrease in concentration was observed for the dimethoate and atrazine, which degraded completely within 64 h, followed by methomyl (80 h), then chlorpyrifos methyl (82 h), and finally the organochlorine heptachlor that needed almost 100 h to completely degrade. These results are in confirmation with previous works for these pesticides in water. Atrazine and dimethoate have been reported to undergo photolysis at 306 nm [32].

**Figure 3:**
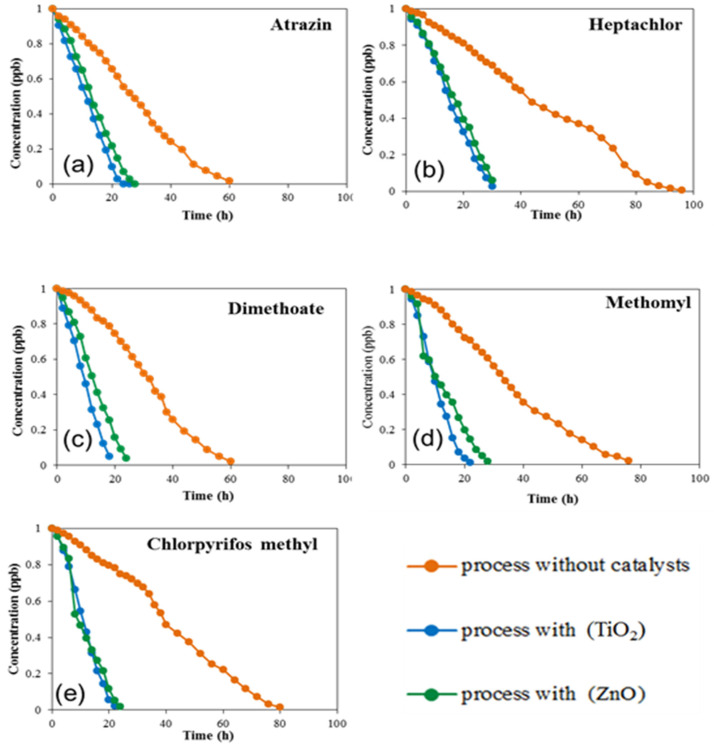
Variation of the concentration of the pesticides as a function of time under UV irradiation in absence of photocatalysts (orange), presence of TiO_2_ (blue) and ZnO (green) for (**a**) atrazine, (**b**) heptachlor, (**c**) dimethoate, (**d**) methomyl, (**e**) chlorpyrifos methyl.

Figure 3 shows the effect of the used photocatalyst on the degradation of the pesticides in the soil. The photodegradation of each pesticide was studied in the presence of 1% of two photocatalysts (ZnO) and (TiO_2_) under UV radiation at a wavelength 306 nm. It can be observed that both photocatalysts greatly enhanced the degradation for all five pesticide residues in comparison with no photocatalyst. These results confirm the beneficial role of using TiO_2_ and ZnO for pesticide degradation in soil. The total initial concentrations for all pesticides were degraded within 20–25 h for all the molecules except the organochlorine heptachlor, which completely degraded after 30 h. Statistical information for the measurements are included in the Supplementary Materials in Table S2 without photocatalyst, Table S3 with 1% TiO_2_, and Table S4 with 1% ZnO. The standard deviation of the values was very low in the range between 0.001–0.004 which confirms the accuracy of the measurement. It is important to mention that it has been previously reported that no degradation for all of the five pesticide residues can occur in absence of sunlight [14]. Even in presence of sunlight, the photolysis of the pesticide residues always occurs at slower rate than in the presence of photocatalyst [32].

**Figure 4:**
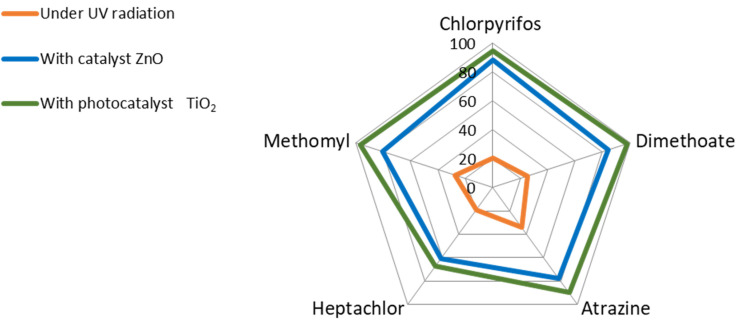
Percentage degradation of the five pesticides after 20 h UV irradiation, without a photocatalyst (orange), with ZnO (blue), and with TiO_2_ (green).

For comparing the effect of the two photocatalysts, the percentage of degradation rate after 20 h irradiation was plotted as shown in Figure 4. It can be clearly observed that the TiO_2_ showed slightly enhanced efficiency over the ZnO especially for the dimethoate, atrazine, and methomyl. The dimethomate is of particular importance since it is a very toxic systemic insecticide [14,33]. Nonetheless, both photocatalysts proved to be suitable for the application of the pesticide removal from soil.

**Figure 5:**
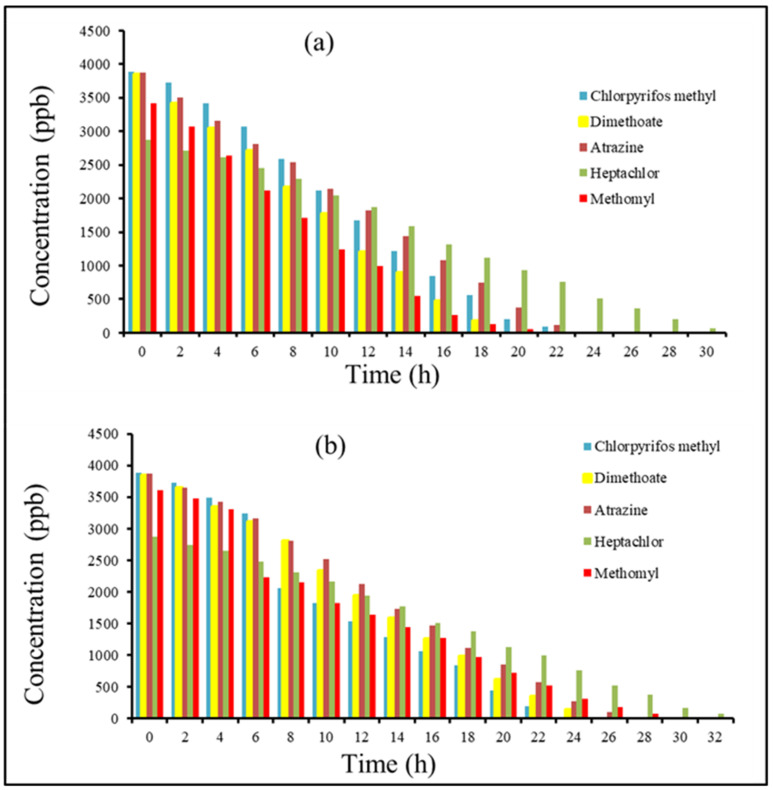
Variation of the concentration of the pesticides as a function of time under UV irradiation in (**a**) presence of TiO_2_ and (**b**) ZnO.

Figure 5 shows a comparison of the concentration of the five pesticides at each time interval. From the figure, it can be shown that starting from the same concentration at around 4000 ppm, the concentrations of organophospates (dimethoanoate and chlorpyrifos methyl) decreased greatly followed by carbamate methomyl, then the organochlorines, the herbicide atrazine, and finally the slowest degradation was with heptachlor.

The trend is similar to photodegradation in absence of photocatalysts, however the degradation time is less. This result is related to chemical structures that affect the photocatalytic degradation process of the pesticide residues in presence of photocatalyst. The pesticides belong to three different chemical classes, i.e., carbamates (methomyl), organophosphates (dimethoate and chlorpyrifos methyl) and organochlorines, which are atrazine and heptachlor. The organophosphates and carbamates are biodegradable and can be degraded under sunlight; however, the organochlorines are persistent. The herbicide atrazine is moderately persistent with a half-life of 66–110 days [34], and heptachlor is almost 2 years [35]. Both pesticides are non-biodegradable and cannot be degraded using light, heat, or microorganisms [36,37]. It is important to mention that understanding the bond energies of the bonds that are cleaved within the degradation process may also explain the differences in the degradation of the pesticides. However, study of the intermediates is outside the scope of our study.

**Figure 6:**
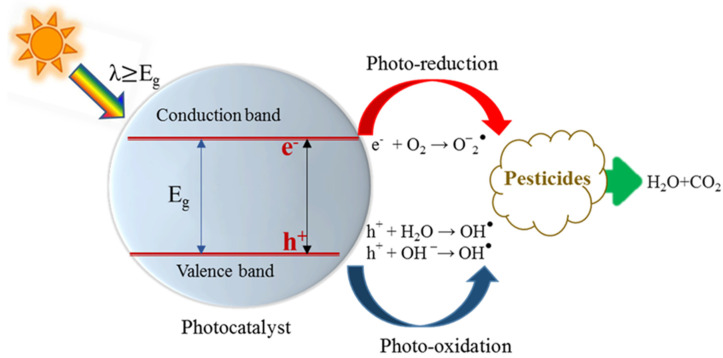
Scheme of photocatalytic degradation mechanism of pesticides on TiO_2__._

To gain more insight into the obtained results, the mechanism of photocatalytic degradation of these pesticides in the soil must be understood. Therefore, it is important to focus on our understanding of the mechanism on the photocatalytic process using the TiO_2_ and ZnO photocatalysts. The photolysis of the pesticides is described elsewhere [32]. The scheme of pesticide degradation on the surface of TiO_2_ is outlined in Figure 6.

Upon irradiation, the semiconductor photocatalyst absorbs electromagnetic UV radiation. The photons with energy equal or greater to its band gap are absorbed by the photocatalyst. For the TiO_2_, UV absorber and the band gaps are typically 3.2 eV for anatase or 3.0 eV for rutile [29]. After light absorption, the photocatalyst becomes excited, i.e., excitons are generated then the electrons are excited from the valence band into the conduction band. (Equation (1)). This process creates photo excited positive holes in the valence band. These excited charge carriers must diffuse though the bulk to the surface of the photocatalyst. If they successfully reach the surface, the holes will oxidize the pre-adsorbed donor molecule (D) into D^+^ and the electrons will reduce the adsorbed acceptors (A) into A- [29,38]. The most common donors are mainly the water H_2_O or hydroxyl ions adsorbed on surface. They get oxidized with the holes to generate the highly reactive hydroxyl radicals (•OH) according to (Equations (2) and (3)) [39]. The excited electrons will reduce adsorbed pesticides on the surface of the photocatalyst. The most common electron acceptor is the O_2_ which gets reduced into the superoxide radical $O_{2}^{\cdot -}$ (Equation (4)) [40]. These generated hydroxyl radicals and superoxide radical anions are the major oxidizing agents that drive the photocatalytic oxidation processes of the pesticide residues. These radicals attack the pesticide molecule and decompose it to mineral ions and other products eventually, i.e., CO_2_ and H_2_O (Equation (5)).

However, it is important to mention that the generated electrons and holes may recombine either at the bulk of the semiconductor due to impurities or low crystallinity or presence of defects at the surface [31]. The recombination of the photo generated electrons and hole with the surface states is in competition with the oxidation/reduction processes due to hole/electron transfer to the adsorbed species on the surface of the photocatalyst, respectively [31]. Therefore, it must be reduced by addition of an electron acceptor such as an oxygen-rich environment, since the efficiency will depend on the oxygen concentration [41,42].

The mechanism is outlined below:

TiO_2_ + hv → TiO_2_ + h_Vb_^+^ + e_cb_^−^, (1)

OH^−^ + h_vb_ ^+^ →●OH, (2)

H_2_O + h_vb_^+^ →●OH + H^+^, (3)

O_2_ + e_cb_^−^ → O_2_^●−^, (4)

TiO_2_ − ●OH _adsorbed_ + RH→CO_2_ + H_2_O, (5)

The pesticides are often degraded through a series of intermediates and proposed pathways that have been investigated in the literature [33,37].

However, since the photocatalyst is in the soil, the surface may not be readily available for the removal of the generated species from the surface. Mass transportation limitations and high resistance hinder the transport of the molecules. Other soil contaminants may also block the active sites through adsorption which may hinder the propagation of radicals and therefore degradation of the pesticide. These are out of the scope of our study and the competition between adsorption and degradation has been studied elsewhere [43].

Nonetheless, it is important to take into consideration factors that affect the photocatalytic degradation of pesticides in soil. These factors include soil thickness [44], irradiation intensity, the moisture [45,46], temperature [47], presence of humic acids, and the physicochemical properties of the soil [48,49]. The moisture is of particular importance and it has been reported that the photocatalytic degradation of pesticides increases as the water content increases by Hilarides et al. [46], Shelton and Parkin [45], Frank et al. [44], and Graebing et al. [47]. This can be explained by the enhancement of formation of hydroxyl radicals and superoxide radical anions (O_2_•^−^) which will enhance the photocatalytic degradation of the pesticides as described above. Another effect of moisture is that water greatly increases the amount of radiation absorbed in the soil [50].

It is important to mention that the properties of the soil are also crucial to determine the environmental impacts of TiO_2_ and the ZnO on the soil. A dual effect is observed for both TiO_2_ and ZnO i.e., they can play both beneficial and toxic roles depending on the soil conditions. Based on the literature, soil properties play a crucial role in the major processes of dispersing, aggregation, stability, bioavailability, and transport of ZnO NPs and their release into the soil [51]. The transfer of ZnO NPs into the soil can affect the soil components, and, consequently, the structure of plants. However, one of the serious side effect of ZnO nanoparticles is that they disturb the bacterial communities in the soil [52]. On the other hand, it has been reported that the use of zinc oxide nanoparticles (ZnO NPs) is beneficial for the soil, and it is expected to increase soil fertility, crop productivity, and food quality [51].

A similar dual effect of TiO_2_ was observed in a recent review. For TiO_2_, a recent review paper showed that adverse side effects were observed in food products where the exposure concentration was very high (>1000 mg/L), which led to accumulation of the TiO_2_ nanoparticles [53]. The TiO_2_ nanoparticles deteriorated the seed germination, growth, and yield in different food crops. Additionally, they possess toxic effects on plant growth-promoting bacteria (PGPB) and decrease the concentration of these bacteria which affects plant productivity, which can be detrimental to soil health [54]. On the other hand, suitable exposure of TiO_2_ concentrations enhanced the germination and growth by increasing the water and mineral uptake from the soil [53]. Other physical properties of the photocatalysts such as the nanoparticle size and shape have been shown to influence the response of soils to TiO_2_.

Future investigations can be complementary studies of these factors to enhance the efficiency of the pesticide photocatalytic degradation while maintaining minimum environmental impact.

## 4. Conclusions

In this work, the photodegradation of the five most abundant pesticides in our samples were studied in ten soil samples taken from two vegetable and date farms in the Saudi Arabia. The samples were extracted by the QuEChERS method and analyzed using gas chromatography–mass spectrometry (GC–MS). The five pesticides with the highest concentrations were degraded under UV radiation at λ = 306 nm in absence and presence of photocatalysts. The photocatalysts used were 1% of TiO_2_ or ZnO. The results showed successful photolysis of atrazine (OCP), chlorpyrifos methyl (OPP), dimethoate (OPP), heptachlor (OCP), and methomyl (Carb) in the presence or absence of the photocatalyst. In the absence of photocatalysts, the pesticide concentrations deteriorated from around 3000–4000 ppb and completely degraded within 60–100 h. However, in the presence of a photocatalyst (TiO_2_ or ZnO), the pesticides degraded at faster rates within 20–24 h, except the heptachlor organochlorine pesticide which needed 30 h. The results show that TiO_2_ exhibited enhanced photocatalytic activity over ZnO. The conditions of the photodegradation process mimic the real environmental conditions and therefore can be applied to soils at a large scale. The effect of the structure of the pesticides on the photodegradation process was reported earlier in previous studies as discussed in aqueous media and not in soil. Investigating the effect of soil on the mechanism of degradation of these pesticides is crucial. Evaluating the effect of moisture, soil thickness, and irradiation intensity gives more insight and allows enhancing the efficiency and recyclability of the photocatalyst. In addition, understanding of the soil properties will aid to assess and minimize the environmental impact of using such nanoparticles. Future work involves studying visible responsive photocatalysts for pesticide degradation.

## Figures and Tables

**Table 1:** Summary of the properties of the photodegraded pesticides [15].

Pesticide Trade Name	IUPAC Name of Active Ingredient	Chemical Class	Use	Entry Route
Atrazine	2-Chloro-4-ethylamino-6-isopropylamino-1,3,5-triazine	Triazine	Herbicide	Systemic
Chlorpyrifos methyl	Cimethoxy-sulfanylidene-(3,5,6-trichloropyridin-2-yl)oxy-λ5-phosphane	OPP	Insecticide	Non-systemic
Dimethoate	*O*,*O*-Dimethyl-*S*-(*N-*methylcarbamoylmethyl) phosphorodithioate	OPP	Insecticide	Systemic
Heptachlor	1,4,5,6,7,8,8-Heptachloro-3a,4,7,7a-tetrahydro-1*H*-4,7-methanoindene	OCP	Insecticide	Systemic
Methomyl	Methyl *N*-(methylcarbamoyloxy)ethanimidothioate)	Carbamates	Insecticide	Systemic

**Table 2:** Gas chromatography mass spectrometry total ion chromatogram (GCMS-TIC) parameters for pesticide analysis method.

Parameter	Conditions	Parameter	Conditions
Carrier gas	Helium, 2 mL/min	Holdup Time	1.3466 min
Inlet temp.	250 °C	Run Time	49.667 min
Mode	Splitless	Solvent Delay	3.00 min
Pressure	9.954 psi	EMV Mode	Relative
Injection Source	GC ALS	EM Voltage	1482
Total Flow	65 mL/min	MS Source	230 °C
Thermal Aux Temp.	281 °C	MS Quad	155 °C
Injection Volume	1 µL	Actual EMV	1482.35
Column	Agilent DB-5 ms 350 °C: 30 m × 250 µm × 0.25 µm	Gain Factor	0.46
Oven Program	Initial temp. 90 °C for 2 min then 6 °C/min to 150 °C for 5 min then 5 °C/min to 220 °C for 5 min then 6 °C/min to 290 °C for 2 min		

## Data Availability

Not applicable.

## Supplementary Materials

The following supporting information can be downloaded online. Table S1: Summary of all the pesticide residues found in 10 soil samples in Al-Kharj district, Saudi Arabia. Table S2: Standard deviation of residual pesticides in the original three soil samples. Table S3: Standard deviation of residual pesticides in the three soil samples with 1% TiO2 catalyst. Table S4: Standard deviation of residual pesticides in the three soil samples with 1% ZnO catalyst.
