# Supplementary material for: Study of the Photocatalytic Degradation of Highly Abundant Pesticides in Agricultural Soils
Source: Molecules. 2022 Jan 19;27(3):634. doi: 10.3390/molecules27030634 (PMC8840474; doi:10.3390/molecules27030634)
Supplement: Supplementary file 1 [file molecules-27-00634-s001.zip › molecules-1530490-supplementary.pdf]

Table S1. Summary of all the pesticide residues found in 10 soil samples in Al Kharj district, Saudi Arabia.

Pesticide Residues Levels in Al kharj Surface Soil Samples (mg/kg = ppm)

Samples #	Farms Type	Chlorpyrifos methyl OPPs	Dimethoate OPPs	Atrazine HERB	Primiphos-methyl OPPs	Chlorpyrifos OPPs	Dicofol OCPs	Methidathion OPPs	Heptachlor OCPs	Endosulfan OCPs	Carbendazim FUNG.	Carbaryl CARB	Ethion OPPs	Diazinon OPPs	Deltamethrin PYR	Cypermethrin PYR	Permethrin PYR	β- Cyfluthrin PYR	Methomyl CARB
**1**	Dates	1.28± 0.011	1.24± 0.015	1.22± 0.023	0.83± 0.009	0.85± 0.012	0.87± 0.011	0.28± 0.003	1.88 ± 0.022	1.85± 0.034	1.86± 0.033	1.78± 0.023	1.79± 0.028	1.81± 0.028	0.31± 0.003	0.33± 0.002	0.34± 0.005	0.79± 0.011	0.73± 0.003
**2**	Dates	2.45± 0.018	2.35± 0.038	2.37± 0.034	0.58± 0.005	0.58± 0.008	0.55± 0.004	0.21± 0.004	0.82± 0.011	0.84± 0.014	0.81± 0.018	0.49± 0.015	0.44± 0.007	0.49± 0.011	0.17± 0.001	0.19± 0.002	0.13± 0.003	1.45± 0.024	1.52± 0.038
**3**	Dates	2.21± 0.021	2.23± 0.022	2.25± 0.022	0.69± 0.007	0.71± 0.011	0.65± 0.005	0.11± 0.003	1.34± 0.019	1.36± 0.023	1.31± 0.015	0.78± 0.018	0.79± 0.012	0.74± 0.009	0.28± 0.003	0.22± 0.003	0.21± 0.002	1.81± 0.038	2.77± 0.033
**4**	Dates	1.71± 0.012	1.62± 0.018	1.73± 0.027	0.44± 0.009	0.43± 0.005	0.46± 0.008	0.27± 0.003	2.01± 0.021	2.85± 0.047	1.89± 0.021	0.88± 0.012	0.85± 0.011	0.86± 0.014	0.21± 0.001	0.18± 0.001	0.19± 0.003	0.91± 0.013	0.95± 0.015
**5**	Dates	3.89± 0.039	3.85± 0.048	3.87± 0.051	1.08± 0.011	1.29± 0.018	1.24± 0.024	0.37± 0.005	2.87± 0.042	1.48± 0.031	1.84± 0.027	0.93± 0.011	0.95± 0.016	0.94± 0.016	0.77± 0.012	0.86± 0.007	0.74± 0.012	1.65± 0.017	3.61± 0.069
**6**	Vegtables	1.44± 0.011	1.48± 0.021	1.49± 0.028	0.49± 0.003	0.42± 0.005	0.46± 0.008	0.17± 0.002	0.69± 0.011	0.71± 0.018	0.68± 0.013	0.56± 0.014	0.52± 0.012	0.54± 0.006	0.16± 0.002	0.16± 0.002	0.17± 0.002	1.67± 0.021	2.62± 0.043
**7**	Vegtables	2.28± 0.024	2.71± 0.019	2.69± 0.031	1.12± 0.014	1.16± 0.023	1.14± 0.017	0.29± 0.002	1.78± 0.018	2.89± 0.044	1.81± 0.019	0.46± 0.018	0.49± 0.017	0.42± 0.012	0.09± 0.002	0.09± 0.002	0.08± 0.001	1.29± 0.024	2.81± 0.027
**8**	Vegtables	2.81± 0.027	2.89± 0.044	2.78± 0.016	1.22± 0.012	1.25± 0.025	1.24± 0.016	0.21± 0.003	2.06± 0.022	1.22± 0.016	1.42± 0.034	0.64± 0.014	0.62± 0.011	0.66± 0.006	0.11± 0.003	0.12± 0.003	0.11± 0.002	1.27± 0.017	2.71± 0.029
**9**	Vegtables	1.22± 0.013	1.22± 0.037	1.26± 0.011	0.44± 0.0017	0.43± 0.004	0.42± 0.005	0.22± 0.003	0.56± 0.013	0.56± 0.018	0.54± 0.012	0.24± 0.004	0.22± 0.006	0.27± 0.003	0.07± 0.001	0.08± 0.002	0.07± 0.003	1.19± 0.031	2.78± 0.018
**10**	Vegtables	1.78± 0.017	1.76± 0.021	1.79± 0.025	0.38± 0.004	0.32± 0.003	0.37± 0.004	0.16± 0.002	1.78± 0.025	1.71± 0.041	1.73± 0.015	0.42± 0.006	0.44± 0.004	0.43± 0.002	0.31± 0.003	0.33± 0.004	0.35± 0.009	1.22± 0.016	1.26± 0.019

Table S2. Standard deviation of residual pesticides in the original three soil samples.

Treatment time /h	Chlorpyrifos	Dimethoate	Atrazine	Heptachlor	Methomyl
0	0.010128535	0.012494	0.013255814	0.0148780	0.019252
2	0.004421594	0.007662	0.008165375	0.0116376	0.009889
4	0.007069409	0.008208	0.00754522	0.0099303	0.008726
6	0.003059126	0.004779	0.00503876	0.0079443	0.006066
8	0.00503856	0.004104	0.00498708	0.0074216	0.006177
10	0.005861183	0.002468	0.003204134	0.0025784	0.004017
12	0.003161954	0.003013	0.003565891	0.0055052	0.004765
14	0.002339332	0.003039	0.005813953	0.0059930	0.006066
16	0.003213368	0.006961	0.006770026	0.0043554	0.008144
18	0.00377892	0.005532	0.003979328	0.0046690	0.004044
20	0.004601542	0.002571	0.004082687	0.0030662	0.005928
22	0.004652956	0.003403	0.002144703	0.0049477	0.006565
24	0.002107969	0.005558	0.005503876	0.0064808	0.004183
26	0.001902314	0.005221	0.002971576	0.0042160	0.002604
28	0.002879177	0.003013	0.004056848	0.0095122	0.003934
30	0.003496144	0.004857	0.002428941	0.0077352	0.004848
32	0.004678663	0.004364	0.00255814	0.0040767	0.00867
34	0.001773779	0.002961	0.004418605	0.0036585	0.005512
36	0.003521851	0.006104	0.005813953	0.0044948	0.003823
38	0.002930591	0.005506	0.004806202	0.0064808	0.004765
40	0.004473008	0.003039	0.003229974	0.0049826	0.00313

44	0.002596401	0.004623	0.00501292	0.0048084	0.005956
48	0.003290488	0.00239	0.00250646	0.0087456	0.003518
52	0.002956298	0.002519	0.002997416	0.0074564	0.004737
56	0.006503856	0.005532	0.001472868	0.0059930	0.00241
60	0.001953728	0.002286	0.001317829	0.0030314	0.003657
64	0.00251928	-	-	0.0074564	0.004958
68	0.003059126	-	-	0.0064460	0.003629
72	0.001825193	-	-	0.0045993	0.004377
76	0.003753213	-	-	0.0097909	0.003906
80	0.001465296	-	-	0.0078049	-

Table S3. Standard deviation of residual pesticides in the three soil samples with 1% TiO_2 catalyst.

Treatment time /h	Chlorpyrifos	Dimethoate	Atrazine	Heptachlor	Methomyl
0	0	0	0	0	0
2	0.000903	0.000912	0.001292	0.000805	0.00089
4	0.000785	0.001431	0.000538	0.000697	0.00167
6	0.001824	0.000937	0.000789	0.001256	0.002079
8	0.002094	0.001039	0.001034	0.001256	0.001575
10	0.001039	0.003931	0.000298	0.000877	0.001394
12	0.003764	0.00105	0.000789	0.001844	0.000973
14	0.002104	0.001039	0.000895	0.000532	0.000973

16	0.001673	0.000687	0.000789	0.002372	0.002091
18	0.00107	0.0003	0.001126	0.001224	0.001394
20	0.000785	0.001587	0.002073	0.000604	0.000973
22	0.001121	-	0.000831	0.001064	0.001946

Table S4. Standard deviation of residual pesticides in the three soil samples with 1% ZnO catalyst.

Treatment time /h	Chlorpyrifos	Dimethoate	Atrazine	Heptachlor	Methomyl
0	0	0	0	0	0
2	0.00068	0.000654	0.000684	0.000877	0.00167
4	0.000903	0.002995	0.001833	0.001064	0.001526
6	0.001028	0.000937	0.000775	0.001788	0.001269
8	0.000826	0.000912	0.00233	0.001064	0.00064
10	0.001285	0.001132	0.000775	0.001319	0.001685
12	0.000927	0.000937	0.001076	0.001064	0.001421
14	0.000647	0.000912	0.001044	0.000201	0.00016
16	0.000771	0.000654	0.003663	0.001597	0.001466
18	0.005545	0.000779	0.002442	0.001408	0.00089
20	0.000973	0.001281	0.000395	0.001224	0.001153
22	0.000771	0.002357	0.001126	0.001919	0.001816
24	0.000514	0.000794	0.001044	0.00112	0.000973
